# Electrochemical Nanocomposite Single-Use Sensor for Dopamine Detection

**DOI:** 10.3390/s19143097

**Published:** 2019-07-13

**Authors:** Giulia Selvolini, Cinzia Lazzarini, Giovanna Marrazza

**Affiliations:** 1 Department of Chemistry “Ugo Schiff”, University of Florence, Via della Lastruccia 3, 50019 Sesto Fiorentino (FI), Italy; 2 Istituto Nazionale Biostrutture e Biosistemi (INBB), Research Unit of Florence, Viale delle Medaglie d’Oro 305, 00136 Roma, Italy

**Keywords:** dopamine, conducting polymer, gold nanoparticles, serum, electrochemical

## Abstract

In this work, we report the development of a simple and sensitive sensor based on graphite screen-printed electrodes (GSPEs) modified by a nanocomposite film for dopamine (DA) detection. The sensor was realized by electrodepositing polyaniline (PANI) and gold nanoparticles (AuNPs) onto the graphite working electrode. The sensor surface was fully characterized by means of the cyclic voltammetry (CV) technique using [Fe(CN)_6_]^4−/3−^ and [Ru(NH_3_)_6_]^2+/3+^ as redox probes. The electrochemical behavior of the nanocomposite sensor towards DA oxidation was assessed by differential pulse voltammetry (DPV) in phosphate buffer saline at physiological pH. The sensor response was found to be linearly related to DA concentration in the range 1–100 μM DA, with a limit of detection of 0.86 μM. The performance of the sensor in terms of reproducibility and selectivity was also studied. Finally, the sensor was successfully applied for a preliminary DA determination in human serum samples.

## 1. Introduction

The understanding of the chemistry of the brain, its structure, functions and, in particular, the neurotransmission process, has been a long-term goal. The brain plays a major role as both an information storage and processing system. Neurotransmission is the process of exchanging and using of this information, and it occurs within a discrete group of highly specialized cells called neurons. Neurotransmitters are substances that aid in transmitting the impulses between the nerve cells, or between a nerve and a muscle, acting as messengers in the synaptic transmission process [1]. They are essential for human health and any imbalance in their activities can cause serious mental disorders. Neurotransmitters are present in various biological fluids, including serum, plasma, platelets, cerebral spinal fluid, urine, and saliva.

Dopamine (DA) is a neurotransmitter belonging to the catecholamine family. DA plays a crucial role in motor coordination, motivational behavior and the regulation of cognitive processes such as attention and working memory. It is involved in reward pathways, which is important in mediating the effects of abusive drugs. DA acts on a range of receptors located in various brain regions and in the periphery. Alterations in the optimal DA concentration have been associated with different neurodegenerative (Parkinson’s) and psychotic (Schizophrenia, and addiction) disorders [2]. Parkinsonian symptoms appear when dopaminergic neuronal death exceeds a critical threshold of 70%–80%. The decreased level of DA is directly associated with an uncontrolled motor function, which leads to an inability in neutralizing the imbalance in neurotransmitters.

Designed electrochemical sensors and micro-sensors have demonstrated a great potential for rapid, real-time measurements with high spatial resolution [3,4,5,6]. Therefore, they can facilitate the study of the role and action mechanism of neurotransmitters. Moreover, they can find potential uses in biomedicine because real-time monitoring of extracellular neurotransmitters concentration offers great benefits for the diagnosis and treatment of neurological disorders and diseases [7,8]. The use of electrochemical sensors for DA determination represents a perfect analytical approach considering their low cost and the short time required for analysis. Moreover, they can be suitable for a routine chair-side test represented by a point-of-care testing (POCT) device. Different strategies have been employed to realize the modification of electrode surfaces for improving the selectivity, sensitivity, and accuracy [9,10,11,12,13,14,15,16].

Over the last few decades, conductive polymers have emerged as an alternative to traditional electrode materials since they combine the electrical and optical properties of both metals and semiconductors. In particular, electrodeposited polymers have several advantages, including the ease of preparation of uniform films with a well-controlled thickness directly onto the electrodic surface [17,18,19,20]. Many conductive polymer systems have been reported; however, polyaniline (PANI), polyacetylene, polypyrrole and poly(3,4-ethylenedioxythiophene) (PEDOT) are the most intensively studied [21]. PANI is popular among organic conjugated polymers because of its ease of synthesis, low cost, uniform conductive mechanism, and superior environmental stability in the presence of oxygen and water [22]. In general, PANI owns a high electrical conductivity only under acidic conditions, due to its redox state associated with the protonation of nitrogen atoms in the polymer backbone. Thus, PANI has a sufficient conductivity at neutral or alkaline conditions. Therefore, since PANI shows peculiar conductive features only in acidic pH values, which makes its application in sensing a big deal, it is often doped with something that can increase these properties, such as noble metal nanoparticles. The incorporation of metal and metal oxides into conductive polymers can enhance electron transfer, thus improving both their conductivity and stability [23]. The obtained composite materials with a well-controlled composition and electrochemical properties provide rapid and accurate sensing due to their selectivity, high sensitivity, many active sites, homogeneity, and strong adherence to the electrode. Specifically, PANI doped with gold nanoparticles (AuNPs) has been already characterized and successfully applied as an electrochemical platform for the biosensing of pesticides in the environmental field [24,25,26].

In this work, we combined the features of a conductive polymer and gold nanoparticles into an AuNPs@PANI nanocomposite film, directly realized onto the graphite screen-printed electrodes (GSPEs) surface, in order to detect DA neurotransmitter. Nanocomposite films involving conducting polymers have already been applied in DA detection. For instance, Zablocka et al. [27] reported the modification of a gold electrode with a polypyrrole-mesoporous silica molecular sieves (MCM-48) nanostructured film, while Ali et al. [28] presented a PANI-carbon nanotubes composite applied via a nonoxidative approach. Many electrochemical approaches with low detection limits for DA have been already presented [29,30,31,32] and most of them make use of glassy carbon, carbon paste [33], or gold electrodes, which are not disposable and whose surface is sometimes difficult to be properly cleaned or regenerated. The novelty of the realized sensor compared to those reported in literature was the use of a fast and easy-synthesizable nanocomposite film coupled with screen-printed electrodes for faster, more sensitive, disposable and cost-effective detection, which are features that could be all suitable in future POCT analysis. In order to assess the suitability of the developed sensor for a possible integration in an easy-to-use kit, and to evaluate the influence of the matrix effect, preliminary experiments were performed in certified human serum samples. 

## 2. Materials and Methods

### 2.1. Chemicals

Aniline (C_6_H_7_N), perchloric acid (HClO_4_), tetrachloroauric acid (HAuCl_4_), sulfuric acid (H_2_SO_4_), dopamine hydrochloride (C_8_H_11_NO_2_∙HCl), di-sodium hydrogen phosphate (Na_2_HPO_4_), sodium di-hydrogen phosphate di-hydrate (NaH_2_PO_4_·2H_2_O), sodium chloride (NaCl), potassium chloride (KCl), potassium ferrocyanide (K_4_[Fe(CN)_6_]), potassium ferricyanide (K_3_[Fe(CN)_6_]), hexaammineruthenium(II) chloride ([Ru(NH_3_)_6_]Cl_2_), hexaammineruthenium(III) chloride ([Ru(NH_3_)_6_]Cl_3_) and human male serum (type AB) were purchased from Merck (Milan, Italy). 

Milli-Q water was used for all solutions. The buffer solution used in this work was 0.1 M phosphate buffer with 0.1 M NaCl pH 7.0 (PBS). 

### 2.2. Apparatus

Electrochemical measurements were carried out with a portable potentiostat/galvanostat PalmSens electrochemical analyzer (PalmSens, The Netherlands), and the results analyzed by PSTrace 5.6 software. 

The sensors were realized using screen-printed electrodes (SPEs) formed by graphite working electrode (3 mm diameter), silver pseudo-reference electrode and graphite counter electrode. The SPEs were purchased from EcoBioServices (Florence, Italy). 

All the reported potentials refer to the pseudo-reference silver screen-printed electrode and all the measurements were carried out at room temperature.

### 2.3. Sensor Development

**Figure 1:**
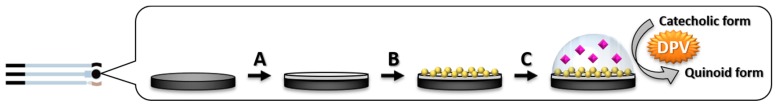
Scheme of the nanocomposite sensor for dopamine detection. (**A**) Electropolymerization of aniline using a 2.5 mM solution in 50 mM HClO_4_ by cyclic voltammetry (potential range: from −400 mV to +800 mV, scan rate: 50 mV/s, 10 cycles); (**B**) electrodeposition of gold nanoparticles using a 0.5 mM HAuCl_4_ solution in 0.5 M H_2_SO_4_ by cyclic voltammetry (potential range: from −200 mV to +1200 mV; scan rate: 100 mV/s, 15 cycles); (**C**) dopamine detection in 0.1 M PBS pH 7.0 by differential pulse voltammetry (potential range: from +5 mV to +600 mV; scan rate: 4 mV/s; step potential: 2 mV; pulse potential: 50 mV; pulse time: 0.05 s).

In this study, an electrochemical nanocomposite sensor for dopamine determination was proposed. As illustrated in Figure 1, the protocol involves the following steps: (a) electropolymerization of aniline onto GSPEs; (b) gold nanoparticles electrodeposition; (c) dopamine determination by DPV measurements. 

#### 2.3.1. Gold Nanoparticles @ Polyaniline Electrodeposition 

The surface of graphite screen-printed working electrodes was first modified by electrodeposition of polyaniline film and gold nanoparticles using cyclic voltammetry (CV). PANI and AuNPs modified GSPEs were realized in accordance with an optimized procedure reported in our previous works [26]. 

Briefly, 50 μL of a 2.5 mM aniline solution in 50 mM HClO_4_ were dropped onto the GSPEs. The potential was cycled from −400 mV to +800 mV for 10 times at 50 mV/s scan rate. The polyaniline-modified GSPEs (PANI/GSPEs) were then washed with 50 μL of a 0.5 M H_2_SO_4_ solution. Then, AuNPs were electrodeposited by dropping 50 μL of a 0.5 mM HAuCl_4_ solution in 0.5 M H_2_SO_4_ onto the PANI/GSPEs. The potential was cycled from −200 mV to +1200 mV at 100 mV/s for 15 times.

The gold nanoparticles/polyaniline-modified GSPEs (AuNPs@PANI/GSPEs) were then washed three times with 50 μL milli-Q water in order to remove excess monomer and free ions from the surface. The sensors were stored at 4 °C in dry conditions for further experiments.

#### 2.3.2. Electrochemical Characterization of the Modified GSPEs

Each modification step of the developed sensing platform was electrochemically characterized by means of CV at different scan rates (25, 50, 75, 100, 125 and 150 mV/s) by dropping onto the SPEs 50 μL of 5.0 mM [Fe(CN)_6_]^4−/3−^ or 1.0 mM [Ru(NH_3_)_6_]^2+/3+^ as redox probes (equimolar solutions in 0.1 M KCl). The potential was scanned from −500 mV to +800 mV for [Fe(CN)_6_]^4−/3−^ and from −550 mV to +50 mV for [Ru(NH_3_)_6_]^2+/3+^, respectively. The current peak height was taken as the electrochemical signal and plotted against the square root of the scan rate (V/s). The obtained curve showed a linear behavior and was fitted by using OriginPro 8.5 software (OriginLab, Northampton, MA, USA) with the Randles-Sevcik equation [34]:

(1) $$i_{p}=0.446nFAC^{0}{\left(\frac{nFvD_{o}}{RT}\right)}^{1/2},$$

where *n* is the number of electrons transferred in the redox events, *A* (cm^2^) is the electrode surface area, *D_o_* (cm^2^/s) is the diffusion coefficient of the oxidized analyte and *C*^0^ (mol/cm^3^) is the analyte bulk concentration.

The nanocomposite sensor (AuNPs@PANI/GSPE) was further characterized by means of CV in the presence of 50 μL of 50 μM DA in 0.1 M PBS pH 7.0 by cycling the potential from −500 mV to +800 mV at different scan rates (25, 50, 75, 100, 125 and 150 mV/s). 

The sensor was designed for single use, thus, after the analysis, the cells were discarded.

#### 2.3.3. Dopamine Detection

The calibration curve was obtained by dropping various dopamine solutions at different concentrations (ranging from 1 to 100 μM) in PBS onto the nanocomposite sensor. DA, as an electroactive compound, was oxidized and detected by means of differential pulse voltammetry (DPV) by scanning the potential from +5 mV to +600 mV at 4 mV/s (2 mV step potential, 50 mV pulse potential, 0.05 s pulse time).

The current peak height was taken as the electrochemical signal and plotted versus dopamine concentration. The obtained curve was fitted by using OriginPro 8.5 software.

#### 2.3.4. Real Samples Analysis

Preliminary experiments for the determination of DA in human serum were also performed. The real samples were diluted at a proper ratio in PBS buffer and then spiked with standard addition of DA. The sensor response was then determined by DPV measurements, under the same conditions used for DA calibration curve.

## 3. Results and Discussion

### 3.1. Modification of GSPEs

**Figure 2:**
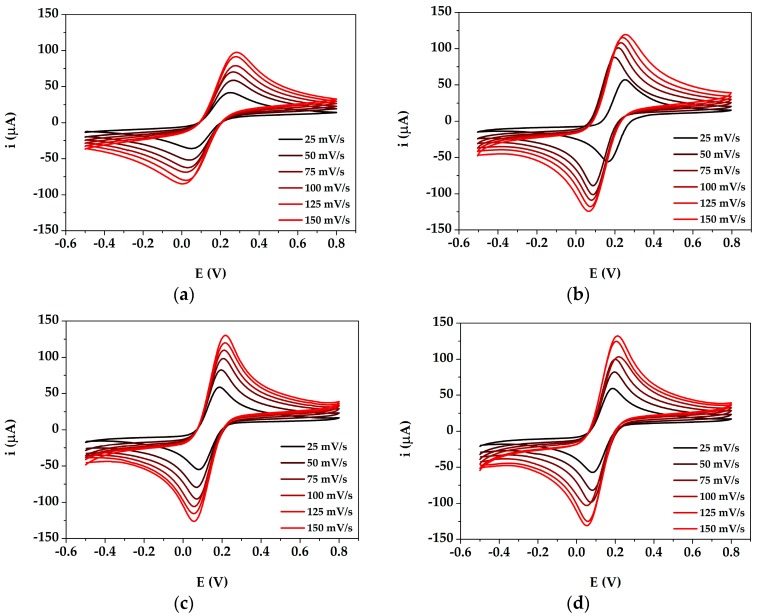
Cyclic voltammograms of bare and modified GSPEs in 5 mM [Fe(CN)_6_]^4−/3−^ with 0.1 M KCl. (**a**) GSPE; (**b**) PANI/GSPE; (**c**) AuNPs/GSPE; (**d**) AuNPs@PANI/GSPE. Experimental parameters: potential range from −500 mV to +800 mV; scan rates 25, 50, 75, 100, 125 and 150 mV/s.

**Figure 3:**
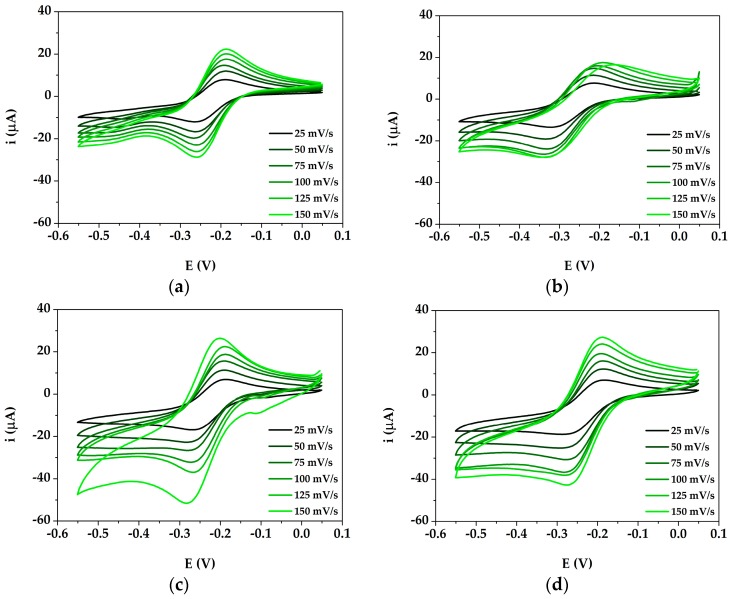
Cyclic voltammograms of bare and modified GSPEs in 1 mM [Ru(NH_3_)_6_]^2+/3+^ with 0.1 M KCl. (**a**) GSPE; (**b**) PANI/GSPE; (**c**) AuNPs/GSPE; (**d**) AuNPs@PANI/GSPE. Experimental parameters: potential range from −550 mV to +50 mV; scan rates 25, 50, 75, 100, 125 and 150 mV/s.

The nanocomposite film was characterized in all its assembly steps by means of CV at different scan rates (25, 50, 75, 100, 125 and 150 mV) in presence of the two differently charged redox probes ([Fe(CN)_6_]^4−/3−^ and [Ru(NH_3_)_6_]^2+/3+^, in order to understand the effect of the charge itself on the interaction of the redox probe with the electrode surface. The cyclic voltammograms obtained for bare and modified GSPEs (PANI/GSPE, AuNPs/GSPE, AuNPs@PANI/GSPE) in [Fe(CN)_6_]^4−/3−^ are shown in Figure 2a–d, while those in [Ru(NH_3_)_6_]^2+/3+^ are shown in Figure 3a–d.

The electroactive surface area was calculated by applying the Randles-Sevcik equation at the angular coefficient of the linear regression reporting the current peak height plotted versus the square root of the scan rate. The obtained area values are reported Table 1 for both the tested redox couples.

Redox peaks were observed at the modified electrodes: PANI/GSPEs, AuNPs/GSPEs and AuNPs/PANI/GSPEs gave higher current response compared to bare GSPE. Thus, the modified electrodes demonstrated a faster charge transport behavior, which was due to an increase of the effective surface area of the electrodes modified with different configurations. The scan rate study shows that both the anodic current (i_pa_) and cathodic current (i_pc_) increased with an increase in the scan rate (25 to 150 mV/s). Regarding the electroactive surface area, a different behavior was observed for both redox probes. In the case of the negatively charged redox couple ([Fe(CN)_6_]^4−/3−^), the value increased following the order GSPEs < PANI/GSPEs < AuNPs/GSPEs < AuNPs@PANI/GSPEs, as the negative charge of the complex is probably being attracted by the positively charged amino groups of PANI polymeric backbone. In the case of the positively charged redox couple ([Ru(NH_3_)_6_]^2+/3+^), the value increased following the order PANI/GSPEs < GSPEs < AuNPs@PANI/GSPEs < AuNPs/GSPEs; even if the use of PANI gave a more reproducible surface, the presence of the polymer established a charge repulsion with the redox probe, which led to a decrease of the electroactive area value with respect to GSPEs and AuNPs/GSPEs.

The electrode surface successfully modified with AuNPs@PANI provided the necessary conduction pathways, besides acting like a nanoscale electrode in promoting electron transfer between the analyte and the electrode surface.

### 3.2. Study of DA Oxidation by Cyclic Voltammetry at AuNPs@PANI/GSPE

**Figure 4:**
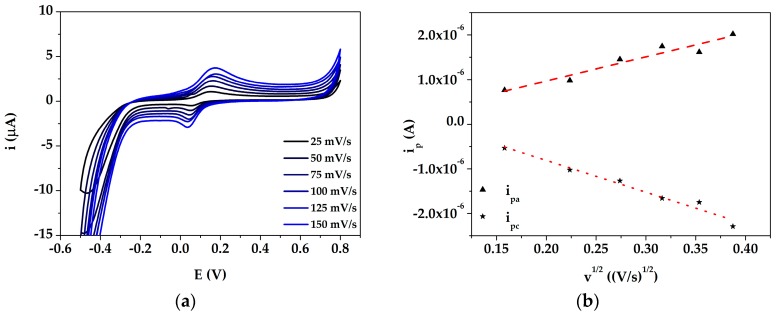
(**a**) Cyclic voltammograms of AuNPs@PANI/GSPEs performed with 50 μM dopamine in 0.1 M PBS pH 7.0 at different scan rates; (**b**) Linear relationship between i_p_ vs. v^1/2^. Experimental parameters: potential range from −500 mV to +800 mV; scan rates 25, 50, 75, 100, 150 mV/s.

Cyclic voltammetry was performed using 50 μM dopamine in PBS at different scan rates (25, 50, 75, 100, 125 and 150 mV/s). The redox peak current height increased with increasing the scan rate from 25 mV/s to 150 mV/s, as shown in Figure 4.

A good linearity was obtained between the redox peak current and the square root of the scan rate with correlation coefficients of 0.93 and 0.97 for i_pa_ vs. v^1/2^ and i_pc_ vs. v^1/2^, respectively. The obtained results suggest that the electron transfer reaction at the electrode surface was controlled by diffusion processes. The linear relationship of the plot confirmed that the nanocomposite film was electroactive, conducting and confined to the surface. Since the developed AuNPs@PANI/GSPEs demonstrated a good electrochemical response towards DA, they were applied for its determination.

### 3.3. Dopamine Calibration Curve

**Figure 5:**
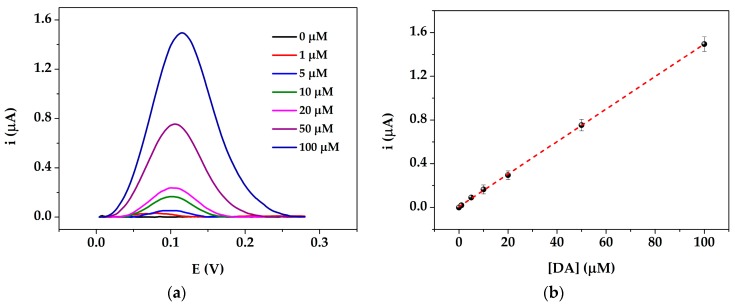
Dopamine detection at AuNPs@PANI/GSPE. (**a**) Differential pulse voltammograms performed with different DA concentrations (0–100 μM) in 0.1 M PBS pH 7.0. Experimental parameters: potential range from +5 mV to +600 mV, scan rate 4 mV/s, step potential 2 mV, pulse potential 50 mV, pulse time 0.05 s; (**b**) Calibration curve for DA. Each point was obtained at least 5 times using different sensors.

A calibration curve of DA in buffered solutions was obtained by DPV technique. An increase of the current peak height was recorded by increasing the DA concentration in the range from 0 to 100 μM (Figure 5, a panel) and a linear relationship was obtained (y = (0.015 ± 9.0∙10^−5^) x + (0.007 ± 0.004)) with a good regression value of 0.998 (Figure 5, b panel). The limit of detection (LOD), calculated as 3.3 S_blank_/slope, was found to be 0.86 μM.

**Figure 6:**
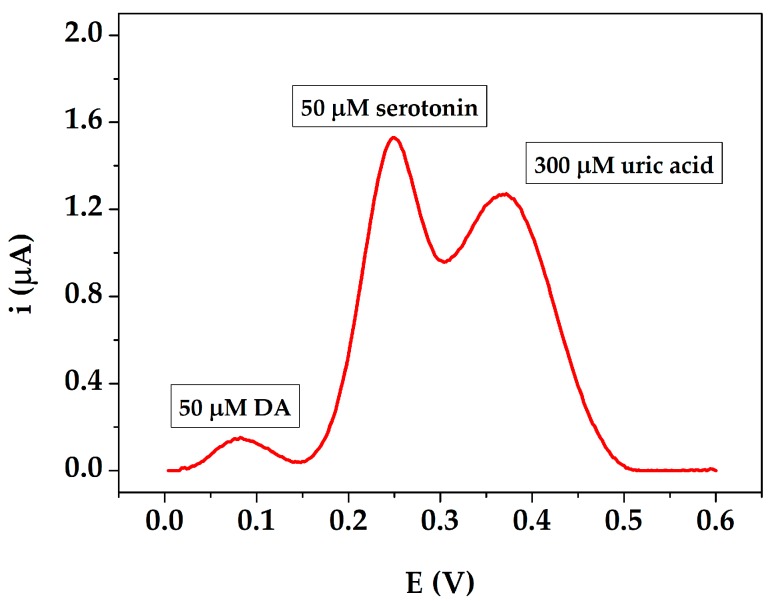
Differential pulse voltammograms performed with 50 μM DA, 300 μM uric acid and 50 μM serotonin in 0.1 M PBS pH 7.0. Experimental parameters: potential range from +5 mV to +600 mV, scan rate 4 mV/s, step potential 2 mV, pulse potential 50 mV, pulse time 0.05 s.

The selectivity of the sensor was investigated by detecting 50 μM of DA solution in presence of possible interfering substances, such as uric acid and serotonin. In non-pathological conditions, the concentrations of uric acid and serotonin are in the micromolar range in serum, for this reason the 300 μM uric acid and 50 μM serotonin solutions were tested. The potential peak values were well separated resulting at +84 mV for DA, +251 mV for serotonin and +367 mV for uric acid (Figure 6). Therefore, DA can be successfully measured even in the presence potential interferents by the AuNPs@PANI/GSPEs sensor. 

### 3.4. Serum Samples Analysis

In order to evaluate the operability of the proposed sensor, some preliminary experiments in human serum samples were performed. With this aim, DA standard solutions were spiked in serum, which was diluted with PBS, without any other pretreatment. The DA response was then determined by DPV measurements, in the same conditions used for the calibration curve of DA buffered solutions.

In order to choose the proper dilution ratio, preliminary experiments were performed by spiking with 50 μM DA the real samples diluted at different ratios and by comparing the obtained signals (*i_sample_*) with that of 50 μM DA in PBS (*i_1_*). The results are shown in Table 2.

**Figure 7:**
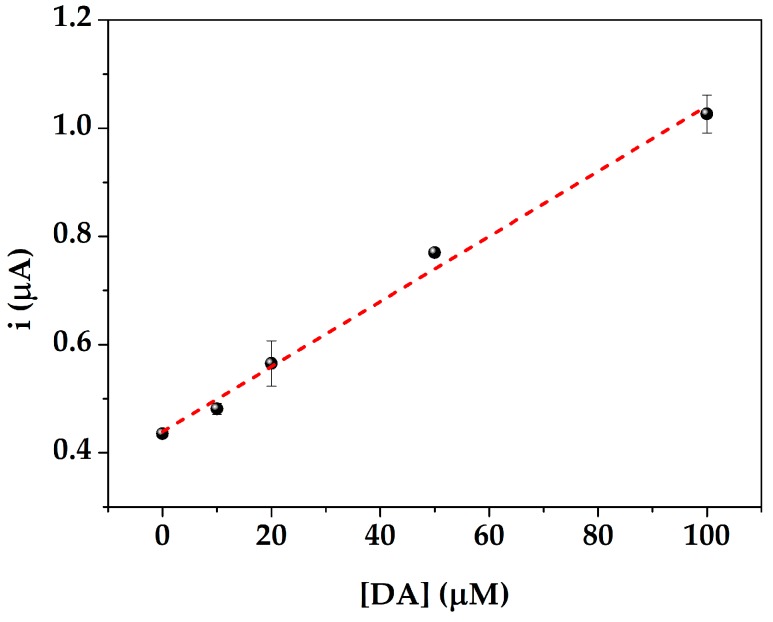
Dopamine detection in 40-fold diluted serum performed with different DA concentrations (0–100 μM). Each point was obtained at least five times using different sensors.

The obtained results showed that by increasing the dilution ratio, the **i_sample_/i_1_** value increases, as the matrix effect was less significant on the response of the sensor. A 40-fold dilution was then chosen. DA was subsequently spiked at different concentrations in the diluted serum samples and a linear calibration curve (y = (0.006 ± 3∙10^−4^) x + (0.439 ± 0.014), R^2^ = 0.992) was obtained (Figure 7).

The %RSD, calculated using at least five measurements with different SPEs, was 2%. These results confirmed the suitability of the use of the proposed nanocomposite sensor for the determination of DA in serum analysis. 

## 4. Conclusions

In this work, we have designed a fast and easy strategy for the modification of GSPEs with PANI and AuNPs for DA electrochemical detection. The nanocomposite sensor facilitates the electron transfer, which leads to an increase in sensitivity towards DA oxidation at the sensor surface. A good linear relationship between the current peak values and the DA concentration in the range from 1 to 100 μM, with a limit of detection of 0.86 μM, was obtained. Good sensitivity and reproducibility were achieved for DA detection, with a linear response that meets clinical needs. The sensor was then preliminarily applied to measure DA in human serum.

Even if in vivo studies should always be performed to test the actual applicability of the device, the easiness of this nanocomposite sensor building procedure combined with the use of a portable instrument confer upon it a great potential to be used as a disposable, cost-effective, and fast device for DA detection in point-of-care analysis.

## Figures and Tables

**Table 1:** Electroactive areas of different platforms (in mm^2^), calculated from the CV scans performed in [Fe(CN)_6_]^4−/3−^ and [Ru(NH_3_)_6_]^2+/3+^ redox probes.

		GSPE	PANI/GSPE	AuNPs/GSPE	AuNPs@PANI/GSPE
[Fe(CN)_6_]^4−/3−^	A_anodic_	6.8	7.8	9.1	9.3
	A_cathodic_	6.2	8.3	8.9	9.2
	Ā	6.5	8.0	9.0	9.2
	%RSD	7	5	2	1
[Ru(NH_3_)_6_]^2+/3+^	A_anodic_	1.8	0.8	2.3	2.1
	A_cathodic_	2.1	1.4	3.1	2.4
	Ā	2.0	1.1	2.7	2.3
	%RSD	10	36	22	8

**Table 2:** Measurements of DA current peak height by varying the dilution ratio of serum samples. The experiments were performed with 50 μM DA in diluted serum samples at different ratios with 0.1 M PBS pH 7.0. Each measurement was repeated at least five times using different sensors.

Dilution Ratio	i_sample_/i_1_	%RSD
1:5	0.10	1.15
1:10	0.07	1.48
1:20	0.16	0.84
1:40	0.23	0.73

## References

[1] Zemková H., Stojilkovic S.S. (2018). Neurotransmitter receptors as signaling platforms in anterior pituitary cells. Mol. Cell. Endocrinol..

[2] Sangubotla R., Kim J. (2018). Recent trends in analytical approaches for detecting neurotransmitters in Alzheimer’s disease. Trends Anal. Chem..

[3] Baranwal A., Chandra P. (2018). Clinical implications and electrochemical biosensing of monoamine neurotransmitters in body fluids, in vitro, in vivo, and ex vivo models. Biosens. Bioelectron..

[4] Bucher E.S., Wightman R.M. (2015). Electrochemical analysis of neurotransmitters. Annu. Rev. Anal. Chem..

[5] Si B., Song E. (2018). Recent advances in the detection of neurotransmitters. Chemosensors.

[6] Xiao G., Xu S., Song Y., Zhang Y., Li Z., Gao F., Xie J., Sha L., Xu Q., Shen Y. (2019). In situ detection of neurotransmitters and epileptiform electrophysiology activity in awake mice brains using a nanocomposites modified microelectrode array. Sens. Actuators B Chem..

[7] Tavakolian-Ardakani Z., Hosu O., Cristea C., Mazloum-Ardakani M., Marrazza G. (2019). Latest trends in electrochemical sensors for neurotransmitters: A review. Sensors.

[8] Emran M.Y., Shenashen M.A., Mekawy M., Azzam A.M., Akhtar N., Gomaa H., Selim M.M., Faheem A., El-Safty S.A. (2018). Ultrasensitive in-vitro monitoring of monoamine neurotransmitters from dopaminergic cells. Sens. Actuators B Chem..

[9] Ramachandran A., Panda S., Karunakaran Yesodha S. (2018). Physiological level and selective electrochemical sensing of dopamine by a solution processable graphene and its enhanced sensing property in general. Sens. Actuators B Chem..

[10] Dinesh B., Saraswathi R., Senthil Kumar A. (2017). Water based homogenous carbon ink modified electrode as an efficient sensor system for simultaneous detection of ascorbic acid, dopamine and uric acid. Electrochim. Acta.

[11] Tsierkezos N.G., Ritter U., Nugraha Thaha Y., Knauer A., Fernandes D., Kelarakis A., McCarthy E.K. (2018). Boron-doped multi-walled carbon nanotubes as sensing material for analysis of dopamine and epinephrine in presence of uric acid. Chem. Phys. Lett..

[12] Ibáñez-Redín G., Wilson D., Gonçalves D., Oliveira O.N. (2018). Low-cost screen-printed electrodes based on electrochemically reduced graphene oxide-carbon black nanocomposites for dopamine, epinephrine and paracetamol detection. J. Colloid Interface Sci..

[13] Diaz-Diestra D., Thapa B., Beltran-Huarac J., Weiner B.R., Morell G. (2017). L-cysteine capped ZnS: Mn quantum dots for room-temperature detection of dopamine with high sensitivity and selectivity. Biosens. Bioelectron..

[14] Yan X., Gu Y., Li C., Zheng B., Li Y., Zhang T., Zhang Z., Yang M. (2018). Morphology-controlled synthesis of Bi2S3 nanorods-reduced graphene oxide composites with high-performance for electrochemical detection of dopamine. Sens. Actuators B Chem..

[15] Maduraiveeran G., Sasidharan M., Ganesan V. (2018). Electrochemical sensor and biosensor platforms based on advanced nanomaterials for biological and biomedical applications. Biosens. Bioelectron..

[16] Zhang S.J., Kang K., Niu L.M., Kang W.J. (2019). Electroanalysis of neurotransmitters via 3D gold nanoparticles and a graphene composite coupled with a microdialysis device. J. Electroanal. Chem..

[17] Moon J.M., Thapliyal N., Hussain K.K., Goyal R.N., Shim Y.B. (2018). Conducting polymer-based electrochemical biosensors for neurotransmitters: A review. Biosens. Bioelectron..

[18] Taylor I.M., Robbins E.M., Catt K.A., Cody P.A., Happe C.L., Cui X.T. (2017). Enhanced dopamine detection sensitivity by PEDOT/graphene oxide coating on in vivo carbon fiber electrodes. Biosens. Bioelectron..

[19] Tertiş M., Florea A., Adumitrăchioaie A., Cernat A., Bogdan D., Barbu-Tudoran L., Jaffrezic Renault N., Săndulescu R., Cristea C. (2017). Detection of dopamine by a biomimetic electrochemical sensor based on polythioaniline-bridged gold nanoparticles. Chempluschem.

[20] Tertiș M., Cernat A., Lacatiș D., Florea A., Bogdan D., Suciu M., Săndulescu R., Cristea C. (2017). Highly selective electrochemical detection of serotonin on polypyrrole and gold nanoparticles-based 3D architecture. Electrochem. Commun..

[21] Cherrington R., Liang J. (2016). Materials and Deposition Processes for Multifunctionality. Design and Manufacture of Plastic Components for Multifunctionality: Structural Composites, Injection Molding, and 3D Printing.

[22] Yilmaz F., Kukukyavuz Z. (2010). Solution properties of polyaniline. Polym. Int..

[23] Saberi R.-S., Shahrokhian S., Marrazza G. (2013). Amplified electrochemical DNA sensor based on polyaniline film and gold nanoparticles. Electroanalysis.

[24] Dakshayini B.S., Reddy K.R., Mishra A., Shetti N.P., Malode S.J., Basu S., Naveen S., Raghu A.V. (2019). Role of conducting polymer and metal oxide-based hybrids for applications in ampereometric sensors and biosensors. Microchem. J..

[25] Rapini R., Cincinelli A., Marrazza G. (2016). Acetamiprid multidetection by disposable electrochemical DNA aptasensor. Talanta.

[26] Selvolini G., Băjan I., Hosu O., Cristea C., Săndulescu R., Marrazza G. (2018). DNA-based sensor for the detection of an organophosphorus pesticide: Profenofos. Sensors.

[27] Zablocka I., Wysocka-Zolopa M., Winkler K. (2019). Electrochemical detection of dopamine at a gold electrode modified with a polypyrrole–mesoporous silica molecular sieves (MCM-48) film. Int. J. Mol. Sci..

[28] Ali S.R., Ma Y., Parajuli R.R., Balogun Y., Lai W.Y.C., He H. (2007). A nonoxidative sensor based on a self-doped polyaniline/carbon nanotube composite for sensitive and selective detection of the neurotransmitter dopamine. Anal. Chem..

[29] Chen X., Li D., Ma W., Zhang Y., Xhang D. (2019). Preparation of a glassy carbon electrode modified with reduced graphene oxide and overoxidized electropolymerized polypyrrole, and its application to the determination of dopamine in the presence of ascorbic acid and uric acid. Microchim. Acta.

[30] Fayemi O.E., Adekunle A.S., Kumara Swamy B.E., Ebenso E.E. (2018). Electrochemical sensor for the detection of dopamine in real samples using polyaniline/NiO, ZnO, and Fe_3_O_4_ nanocomposites on glassy carbon electrode. J. Electroanal. Chem..

[31] Filik H., Avan A.A., Aydar S. (2016). Simultaneous detection of ascorbic acid, dopamine, uric acid and tryptophan with Azure A-interlinked multi-walled carbon nanotube/gold nanoparticles composite modified electrode. Arab. J. Chem..

[32] Muratova I.S., Mikhelson K.N. (2018). Voltammetric sensing of dopamine in urine samples with electrochemically activated commercially available screen-printed carbon electrodes. Int. J. Biosens. Bioelectron..

[33] Raoof J.B., Kiani A., Ojani R., Valiollahi R. (2011). Electrochemical determination of dopamine using banana-MWCNTs modified carbon paste electrodes. Anal. Chim. Actaytical Bioanal. Electrochem..

[34] Elgrishi N., Rountree K.J., McCarthy B.D., Rountree E.S., Eisenhart T.T., Dempsey J.L. (2018). A practical beginner’s guide to cyclic voltammetry. J. Chem. Educ..

